# Outdoor malaria transmission risks and social life: a qualitative study in South-Eastern Tanzania

**DOI:** 10.1186/s12936-018-2550-8

**Published:** 2018-10-29

**Authors:** Irene R. Moshi, Lenore Manderson, Halfan S. Ngowo, Yeromin P. Mlacha, Fredros O. Okumu, Ladislaus L. Mnyone

**Affiliations:** 1 Environmental Health and Ecological Sciences Department, Ifakara Health Institute, Kiko Avenue, Mikocheni, PO Box 78373, Dar es Salaam, United Republic of Tanzania; 2 School of Public Health, Faculty of Health Sciences, University of the Witwatersrand, Parktown, Johannesburg, South Africa; 3 Swiss Tropical and Public Health Institute (Swiss TPH), Basel, Switzerland; 4 University of Basel, Basel, Switzerland; 5 Sokoine University of Agriculture, Pest Management Centre, P.O. Box 3110, Morogoro, Tanzania; 6 Institut de Recherche en Sciences de la Santé, Bobo-Dioulasso, Burkina Faso; 7 Institute of Biodiversity, Animal Health and Comparative Medicine, University of Glasgow, Glasgow, G12 8QQ UK

**Keywords:** Community gatherings, Life course events, Kilombero Valley, Outdoor-mosquito bites, Malaria transmission, Vector control, Tanzania

## Abstract

**Background:**

Behaviour changes in mosquitoes from indoor to outdoor biting result in continuing risk of malaria from outdoor activities, including routine household activities and occasional social and cultural practices and gatherings. This study aimed to identify the range of social and cultural gatherings conducted outdoors and their associated risks for mosquito bites.

**Methods:**

A cross-sectional study was conducted in four villages in the Kilombero Valley from November 2015 to March 2016. Observations, focus group discussions, and key informant interviews were conducted. The recorded data were transcribed and translated from Swahili to English. Thematic content analysis was used to identify perspectives on the importance of various social and cultural gatherings that incidentally expose people to mosquito bites and malaria infection.

**Results:**

Religious, cultural and social gatherings involving the wider community are conducted outdoors at night till dawn. Celebrations include life course events, religious and cultural ceremonies, such as Holy Communion, weddings, gatherings at Easter and Christmas, male circumcision, and rituals conducted to please the gods and to remember the dead. These celebrations, at which there is minimal use of interventions to prevent bites, contribute to individual satisfaction and social capital, helping to maintain a cohesive society. Bed net use while sleeping outdoors during mourning is unacceptable, and there is minimal use of other interventions, such as topical repellents. Long sleeve clothes are used for protection from mosquito bites but provide less protection.

**Conclusion:**

Gatherings and celebrations expose people to mosquito bites. Approaches to prevent risks of mosquito bites and disease management need to take into account social, cultural and environmental factors. Area specific interventions may be expensive, yet may be the best approach to reduce risk of infection as endemic countries work towards elimination. Focusing on single interventions will not yield the best outcomes for malaria prevention as social contexts and vector behaviour vary.

## Background

Between 2000 and 2015, global malaria morbidity and mortality declined by 41% and 62%, respectively [1, 2]. This reduction has largely been associated with the high coverage of frontline vector control measures, such as long-lasting insecticide-treated nets (LLINs) and indoor residual spraying (IRS) [2], and the widespread use of the malaria rapid diagnostic test (RDT) for prompt diagnosis and increased access to treatment [3–5]. Social marketing programmes and substantial global investment have improved universal coverage of bed nets and in turn increased personal and community protection [6–9]. Despite these achievements, the burden of malaria remains unacceptably high, with an estimated 216 million cases in 2016 worldwide, 4 million cases above that of from 212 million cases in 2015 [10].

In sub-Saharan Africa (SSA), which suffers the largest malaria burden, malaria prevalence decreased from 17% in 2010 to 13% in 2015 [1]. Tanzania recorded reduction in under-five mortality from 112 deaths to 67 deaths per 1000 live births between 2004 and 2010 [11], and in Muheza district in Tanga region, malaria incidences decreased by 75% between 2000 and 2015 [12–15]. Continued malaria cases and deaths, despite the wide coverage of LLINs and IRS, is associated with insecticide resistance [16–18], decrease in bed net use [19], and changes in mosquito biting behaviour and patterns from indoor biting to early outdoor biting, therefore reducing their contact with insecticide treated surfaces [20–23]. However, other factors that contribute to increasing outdoor feeding includes climate change [24], human behaviour and land use [25], environmental change [26], ecology and increasing zoophagic vectors may all contribute to continued transmission [27–30]. Previously outdoor biting was not considered as important for allocation of interventions because of its low impact on malaria transmission [31, 32], but the current increasingly outdoor biting by *Anopheles arabiensis* and *Anopheles funestus* [33–35] is contributing to continued malaria rates, frequent infections and inhibiting global malaria elimination efforts [33, 36, 37]. Studies have identified the need to address the risks of outdoor malaria transmission [38, 39]. Despite the reported changes in vector’s biting and resting behaviours [40–45], in Tanzania, control efforts have neither attended the outdoor transmission segment nor consider the role of social and cultural factors in malaria transmission [46–50].

Malaria prevalence in Kilombero Valley is about 14% from a study done in 2011 [51]. The major malaria vectors includes *An. arabiensis*, *An. funestus* and *Anopheles gambiae* sensu stricto [34] whereby *An. arabiensis* is the dominant vector for the outdoor settings. The prevalence in this valley might be a result of not only indoor but also outdoor transmission risks, therefore other factors including host-seeking behaviour needs to be well understood to prevent malaria rebound. While behavioural factors are important for individual and household level prevention, a range of social and cultural factors are implicated in malaria transmission and effective interventions use outside human dwellings, that may impact health and well-being of the communities. A study by Dunn et al. [52] has indicated that, minimal bednet use among children in Kilombero Valley is a result of sleeping arrangements within the households thus continue to put this vulnerable group at risks of malaria infection. So, human behaviour and practices that contribute to outdoor malaria transmission risks needs to be well understood for identification and allocation of appropriate intervention to prevent mosquito bites and control malaria. These factors include; local knowledge and perceptions, cultural beliefs and norms, behaviours and practices [53]. This study aimed to identify and explore the significance of social and cultural and practices, and their contributions to exposure and existing outdoor malaria transmission, biting experiences and intervention use in Kilombero Valley.

## Methods

### Study area

**Fig. 1:**
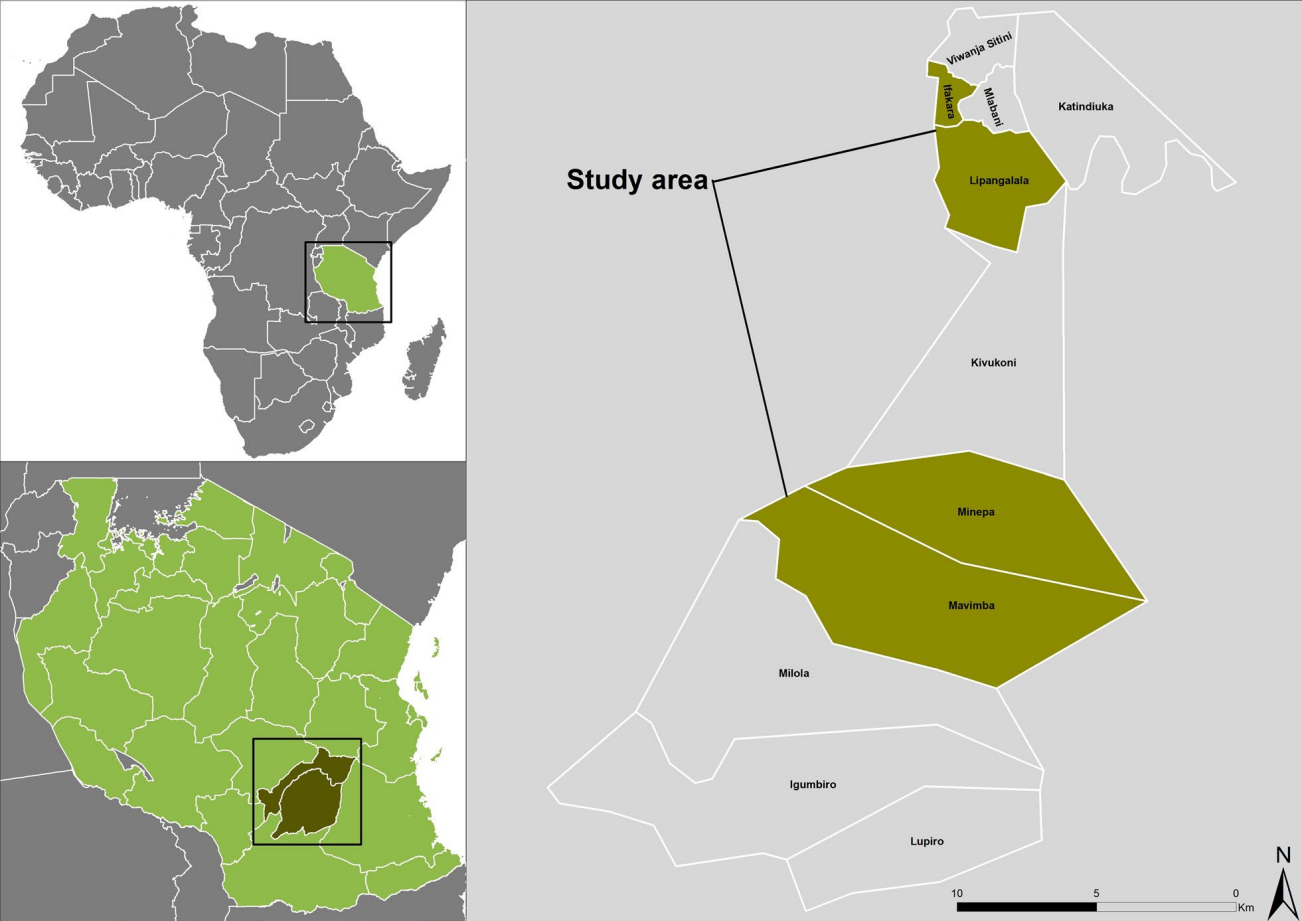
Map of study villages. The study was conducted in both Kilombero and Ulanga Districts

This study was conducted from November 2015 to March 2016 in Kilombero Valley, Morogoro region, South-Eastern Tanzania. The valley is approximately 300 metres above sea level, and experiences a short rainy season from late November to January and a long rainy season from mid-February to May. Annual rainfall ranges from 1000 to 1400 mm; annual temperature ranges from 16 to 32 °C [54]. The study was conducted in Lipangalala and Ifakara Town, Kilombero district, and Minepa and Mavimba, Ulanga district (Fig. 1). The main economic activities of people in the area are rice farming and fishing, and in the semi-urban settings of Lipangalala and Ifakara Town, also small-scale businesses. The majority of houses are made with mud bricks walls, with corrugated iron or thatched roofs and small windows that are rarely covered with insect screens [55].

### Study design

A descriptive cross-sectional study was conducted using focus group discussions (FGDs), key informant interviews (KIIs) and direct observations. The FGDs stimulated recall and encouraged people to share experiences and opinions, which helped to understand the existing interactions, practices, and opinions on social and cultural practices within the study communities. To complement the FGDs data, KII were conducted to further probe into community practices, the meanings attached to them and their importance to the communities. Participants for both FGDs and KIIs were also provided an opportunity to provide recommendations for malaria control. Grounded theory principles guided the study, with theories developing from collected data to generate explanations of relationships and events [56, 57]. In the final stages, thematic analysis was adopted.

### Study participants

Purposive sampling was used to select all study participants. FGD participants were selected from community members, with the following inclusion criteria: 18 years or older; either a caregiver or another adult from a household with at least one child below 5 years of age; voluntary agreement to participate in the study. Eight FGDs (two from each study village) were conducted, with 9–11 people per group (total 45 men, 43 women). All groups included both men and women from two ethnic groups such as Ndamba and Pogolo. The similarity to the ethnic groups adhere to the suggested similar characteristics of the FGD participants [58] as among the factors but the groups were mixed with both sex to stimulate discussion, improve the quality of the discussion, gain comprehensive information on local cultural and social practices within the community and the meanings attached to them [59]. For the KIIs, eight participants (four men, four women) were selected from among village leaders in the four study villages, who were born in the Kilombero Valley or lived in the study villages for at least 10 years.

### Data collection

For both FGDs and KIIs, collected data included demographic variables and information on social and cultural gatherings, exposure to mosquitoes, and malaria prevention. All FGDs and KIIs were conducted by the first author in Swahili, the official and widely spoken language. Voluntary informed consent was obtained from each study participants before data collection, including consents for audio recording and note-taking. Venues for the FGDs and KIIs were selected from the study villages according to accessibility and availability. The venues were mostly the primary school classrooms within the study villages for FGDs while for KII were mostly conducted in the offices or homes of the respondent. Preliminary data analysis took place as FGDs and KIIs continued, allowing for iteration and determination of quality and range of data. Data collection ceased with saturation, when new information were not obtained from both FGDs and KIIs.

### Data processing and analysis

All audio recordings from the FGDs and KIIs were fully transcribed and translated from Swahili to English, and were double checked by comparing the recordings, Swahili and English transcripts to ensure accuracy. Thematic analysis was conducted to identify themes and subthemes [60], with NVIvo software version 13 used to arrange and connect the themes and subthemes [61]. The themes and subthemes included the range, purpose and meanings of social and cultural gatherings, the timing of events, exposure to mosquito bites, and preventive measures, with probing as these themes emerged during data collection. These dominant themes informed the analysis process. Consensus was established where there were contradictions in themes and subthemes through discussion and in relation to the significance of the theme to the research question, community, and people’s health. No contradictions emerged about conceptual issues.

## Results

A total of 96 people participated in the study (88 in FGDs; eight KII respondents). Their distribution is provided in Table 1.

**Table 1:** Distribution of FGD and KII respondents by age and sex

Village name								
Distribution of respondents for FGDs								
Age group	Lipangalala		Ifakara town		Mavimba		Minepa	
	Male	Female	Male	Female	Male	Female	Male	Female
20–29	5	3	3	2	2	3	2	4
30–39	2	1	4	2	4	2	6	4
40–49	3	4	0	5	2	4	3	2
50–59	2	3	3	0	3	2	1	0
Total	12	11	10	11	11	11	12	10
Age distribution of respondents for the KIIs								
All	50	45	72	65	52	49	39	47

### Socio-cultural gatherings

Gatherings and celebrations conducted in the study area fell into three primary categories: religious gatherings, cultural celebrations, and other social celebrations.

### Religious celebrations

These celebrations were associated with baptism, Holy Communion, confirmation and weddings, and among Islam, Mawlid (the birth of Muhammad). These celebrations were generally planned to coincide with the post-harvesting period. The relevant religious ceremony is normally held indoors in a church, but this is followed by celebrations outdoors at the home of the responsible family. A few celebrations were reported to take place in rented venues, but these are relatively scarce, and have open eaves providing minimal or no protection from mosquito bites.

Both Christianity and Islam are practiced in this communities, so adhering to religious ceremonies provides people with a strong sense of identity among Christians. Weddings provide newly married couples with identity within their local community but also in the religious community, while also fulfilling the social expectations. Adults from 20 years and above are expected to get married and religious weddings are especially valued in families as an affirmation of faith as well as social status.

### Cultural gatherings and practices

These gatherings, life course events include funeral and mourning gatherings, children’s initiations, and remembering the dead. Respondents indicated that children’s initiation ceremonies were held for both boys and girls. Initiation for boys may take place any time between the ages of 7 and 17, and involved circumcision and teaching conducted by the *Ngariba* (the person who conducts circumcision/circumciser). At the time of the research, two types of boys’ initiations were conducted, the traditional circumcision and initiation that is mostly practiced in rural Ulanga district, and hospital-based circumcision in urban Kilombero district. Boys’ traditional circumcision (*Jando*) is normally conducted in the forest without anaesthesia. *Jando* not only base on circumcision, but also involves teachings on how to behave well and the responsibilities of the man in the society [62, 63]. Their stay in the forest is often extending over a period of 2 weeks or more and while in the forest, boys are exposed to mosquito bites, and in most cases, no protection against mosquitoes is used. As one KII then explained, “after coming from *jando,* people prepare for a celebration known as *mtoto katoka sunna,* so people such as family members and neighbours, cook, eat and celebrate” (KII respondent). In contrast, in semi-urban settings of Kilombero district, hospital-based circumcision is increasingly practiced, after which the boys are kept inside their homes to heal. Since they do not participate in forest initiation ceremonies, their risk of exposure to mosquito bites is reduced. However, the hospital-based circumcision is undertaken when children are still young from 6 months to 5 years of age, so the teachings may not be provided and if provided my not be of the level of what is taught at traditional initiations.

For girls, initiation involved teaching only, which is undertaken by a respected senior woman in the family or community such as an aunt who is known as *kungwi (*whose main tasks is to teach girls responsibilities of a woman, how to take care of the house and a man as they are then considered to be old enough to start a family, i.e. soon after first menstruation*)*. The initiation of girls signifies that they have reached adulthood, usually after their first menses and normally kept secret. During such occasions, a group of girls stay in the same household for several days and taught about hygiene, self-care, and sexual behaviour with men, especially after marriage.

The initiations of both boys and girls are followed by commemorative celebrations (*kumtoa sunna* which means “Sunnah” to this society means is the celebration after cleansing the child, which involves prayers and taking the child out of the house after the initiation to celebrate with others [64]) organized by parents and guardians and usually conducted outdoors from day through night and until dawn. The congregation may sometimes consist of more than a hundred people who sit outside celebrating. People recognize the possibility of mosquito bites at such occasions, as one FGD participant noted:

In Ifakara, people have adopted traditions from the *Zaramo* people from the coastal areas. “*Ooh. My daughter has grown, ooh, I have circumcised my child, so I will prepare a sunna (Sunnah) a huge celebration. There are other normal celebrations such as wedding receptions (and) child baptism … in all these celebrations, mosquitoes get a chance to bite you/people.”* (Respondent, FGD).

Death provides another occasion for communal gathering. As elsewhere in Tanzania, respondents believed that burying people in their homeland is important to ensure they rest in peace where they belong, and this is portrayed as a way of respecting the deceased and fulfilling their wishes. Death can occur any time, regardless of season and people are expected to provide social support to the bereaved family. As one participant in an FGD reflected, “*the importance of funerals is that there is “God’s will,” so “kumsitiri mwenzetu” (helping each other during the tough times and do the things as the deceased wishes) is among the ways that we can help our fellow to rest in peace.”* (Respondent, FGD).

Since funerals are rarely timed to the post-harvest period, families of the deceased receive financial assistance from relatives, friends and other community members. Most mourners are women, offering their condolences to the relatives of deceased and performing other activities such as cooking, singing religious songs (*zikiri*) throughout the night, and dancing to please the Gods. Although the deaths of infants or stillbirths are not accompanied by extended celebrations, a simple burial will be attended by the mother and close relatives.

Although respondents had different opinions on the importance and significance of conducting various gatherings and ceremonies, their presence at funerals was seen as maintaining a sense of unity and support among each other; and failure to attend would lead the bereaved to feel isolated from others within the village. For funerals, people also felt it was important to stay overnight at or outside the house of the bereaved family as an expression of support. During these gatherings, the mourners who can be relatives, friends, and neighbors sit outdoors, where they exchange stories, chat and reminisce, sing and pray rather than sleep. While they are outdoors, they are frequently bitten by mosquitoes. There is very minimal use of interventions, although occasionally people use repellents or wear long sleeve clothes to prevent bites. Respondents indicated that use of bed net in an outdoor setting was difficult, but also that it would be considered disrespectful, suggesting lack of compassion to those who had lost their loved one:

“*You cannot come with a net into the house when people are mourning. It suggests you think you are better than the rest of the people who are there*” (Respondent, FGD).

Ceremonies for remembering and pleasing the dead are normally conducted 1 year after a death. These are believed to have cultural significance and are considered to be of great importance. These ceremonies are well prepared, and community members believe that such events are necessary to enable the dead to rest in peace and to prevent misfortune. The ceremonies involve visiting and cleaning the grave, creating a tombstone, and rituals such as particular prayers and chants. Afterwards, people spend time with the bereaved family, providing food to eat, drink and talk. These celebrations are always conducted in the post-harvest period, when people have sufficient food and money from selling their harvest and they are conducted outdoors from day time till dawn, often extended for several days depending on the financial capability of the host. During the post-harvest periods people have less farm work, so ensuring that attendance at such events is optimal. Again respondents reflected on exposure to mosquito bites, as one key informant explained:

“*There are these rituals when people gather to celebrate for two days, they cook food, make a local brew, eat and drink together and celebrate. They also pour some local brew on the ground in the belief that they are giving it to their ancestors. These celebrations cannot be done in one day and finished, so most of the time people celebrate for two days. The first day they gather, eat and drink, celebrate the whole night, then in the morning they finalize the celebrations, then celebrate for the second day and finish the following day, and people do not use any prevention from mosquito bites”* (Respondent, KII).

“*These gatherings are mostly in the dry season because it is the time when people are idle or do not have much to do because they are back from harvest; they are just at home and do not go to the farm anymore, so they may just come up with an idea to do a ceremony. Except for gatherings like funerals and mourning, they can happen anytime”* (Respondent, FGD).

In rare cases too, members of the study communities may meet for a certain length of time for family or community issues that require extended discussion and collective decision-making. These discussions too are conducted outdoors in the evening, and mostly preceded by having drinks and foods. Such issues may relate to security and safety, or important government communications, and may be held at any time in the year.

### Local economics

The majority of respondents emphasized that the ceremonies were dictated by various personal interests or motivations. Although such gatherings varied in time, place and duration according to the event celebrated, individual interests such as prestige, material, and financial gains were important reasons that motivated hosting or participating in a gathering. Most women in the study communities belonged to various Village Community Bank Groups (VICOBA), to which they contributed a set amount of money on a monthly basis. Group members supported each other in good and hard times, so when a member hosts a ceremony or gathering, other group members attend and provide gifts to the host. As a result, the hosts gain materially from a ceremony such as; clothing and African print cloth (*vitenge)*; household utensils like glassware and dinner sets; and money. During these celebrations (mostly religious events), the majority of supporters and attendees are women and children, but in mourning and funerals children are less comparing to other events.

Agriculture is the main economic activity of people living in Kilombero Valley, with most people engaged in small-scale agriculture for subsistence and small cash returns. Many people depend on their capacity to borrow both personally or from financial institutions to maintain and cultivate their farms then repay after harvest. Most farmers use traditional methods of cultivation, with timing tied to the rainy season. Farming allows people to get by few months after harvest, therefore, purchasing mosquito repellents is less a priority than food and paying school fees. People weigh what is important to them based on the circumstances, and poverty impacts on the use of intervention for malaria prevention in these settings.

“*Farmers do not have the ability to buy all the agricultural inputs. As a result one has to borrow them from suppliers/shop owners where they return with interest. Then, yes they got the produce like 50 bags of rice, but they borrowed money for farm preparation and weeding so, after returning all these money, they remain with very little and sometimes remain with five bags for consumption. Still, this person expects to farm the following year, and they do not have any other source of income, so they depend on selling the remaining bags to solve all their problems and meet their needs, such as school fees and medications. It reaches a point that someone does not have any money, so he cannot buy repellent instead of food.”* (Respondent, KII)

Respondents had mixed views about the overnight celebrations. Some supported their continuation due to the importance of these celebrations in bringing community members together to enhance social bonds. Others did not appreciate their importance and felt they contributed to the risks of being infected by diseases:

“*Mosquitoes bite people when they are in gatherings like in places of worship, such as mosques and churches, because they bite people when they are settled and not on the move like dancing or the like. They also bite people who are mourning because when people get there, they mostly sit down and sleep without protection, hence providing the best opportunity for mosquitoes to bite.”* (Respondent, FGD).

Most respondents were aware of interventions that repelled mosquitoes when they were outdoors, such as topical repellents and mosquito coils, which they suggest should be freely provided to the communities by the government, but they rarely use them, primarily because of their cost. The most common means of protection that people use are fanning and slapping themselves. A few respondents suggested that some people protected themselves from mosquito bites by wearing heavy pants and long-sleeved tops, but as they pointed out, mosquitoes bite anywhere on the body, mosquitoes could bite through light cloth, protection depended on the thickness of the fabric, and in high temperatures, is was uncomfortable to wear heavy clothing.

Respondents held diverse views about the feasibility of interventions during social and cultural gatherings and ceremonies. Although they acknowledged the need for interventions that could be used to minimize outdoor mosquito bites and possible infection, they also emphasized the need to educate women on the risks of malaria while outdoors, likely associated with women’s predominance in various ceremonies, their exposure to bites on an everyday basis when preparing evening meals, and assumptions about their responsibility for infants and children. Further, probably influenced by the association of the first author with the research institute in Ifakara, people emphasized that government should work hand in hand with Malariologists and research organizations to regularly update the community on disease transmission trends and vector behaviour. One FGD participant stated that:

*“I’d put more emphasis on organizations, because you as an organization, you mainly focus on this sector. I would advise you to go back and speed up the research on this disease to get the solution. That is my main suggestion because we have been struggling for a very long time.”*(Respondent, FGD)

Respondents also reiterated their ability to purchase repellents depending on relative poverty and priorities, and several factors hindered the introduction and sustainability of interventions:

*“I suggest that wadau (stakeholders) for malaria, like organizations and government, should provide interventions such as repellents at affordable rates or even free of charge so that people can use them during gatherings. It is obvious that people will rate (accept) the need to buy this repellent lotion, but when comparing the price of buying it with food and salt, due to uchumi (economic situation), they buy food and not repellent, but if they are provided free then people would use them.”* (Respondent, FGD).

## Discussion

Community gatherings and celebrations are viewed as important, reflecting social norms and local traditions, and providing participants with mutual support, opportunities for socialization and community engagement. Life cycle events, including traditional initiation ceremonies, baptisms and weddings, are vital moments in the socialization of young people, providing sexual education, guidelines regarding gender norms, and norms and expectations as adult members of kinship networks and the community. The support that people provide each other over time at funerals and through an extended period of mourning, and at celebrations such as baptisms, weddings and the prophet’s birthday, are given high priority. In addition, sleeping at the houses of bereaved villagers, helping each other financially and during preparation of events help build and maintain social capital and inclusion. The beliefs that sustain these events reflect strong understandings of what is the “right way” to do things, and the value of reciprocity and collective action.

The life cycle events, celebrations and gatherings around death, and intermittent community gatherings related to government policy and local politics and economics, provide personal satisfaction as well as benefitting the group [65]. There is, as noted above, both tangible benefits and prestige value to hosts when they initiate a celebration, and those who host an event for a long period than others, extending for more than 2 or 3 days, are evidently wealthier and able. People compare and contrast themselves and others in terms of largesse and power, and in this context, are likely to spend money to host a party while they cannot afford to purchase repellents, which might protect them from mosquito bites. At the same time, the amount of money spent on ceremonies and celebratory gatherings is reciprocated over time. Celebrations that are conducted outdoors were reported as associated with mosquito bites, but the social significance and consequences of participating in such events outweigh the importance of using interventions while outdoors. However, similar findings on the importance of community social norms against intervention use was also observed in Uganda where net use during community gatherings outdoors was observed to indicates ones feel proud [66].

**Fig. 2:**
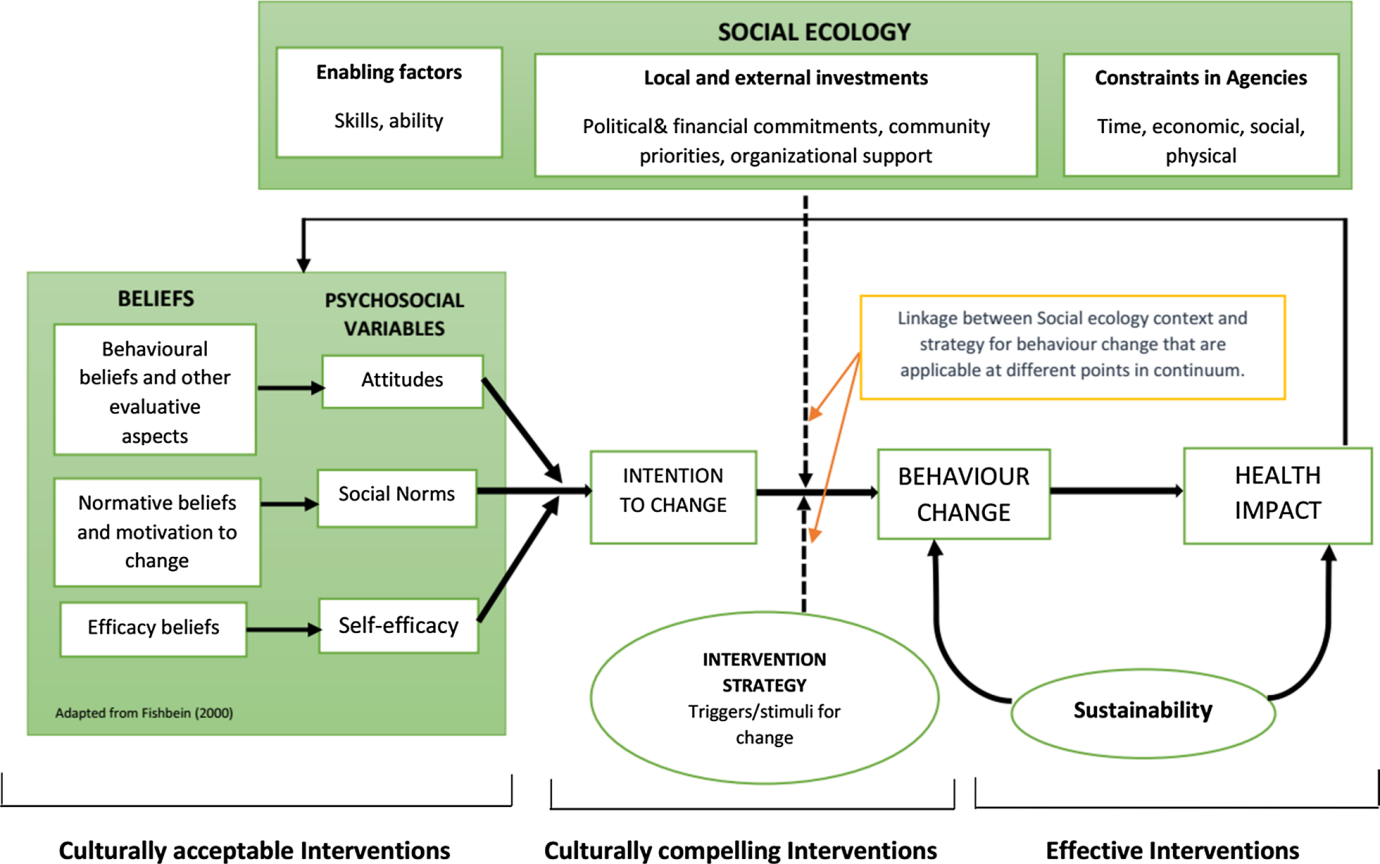
Diagram indicating the social ecology model of behaviour change, for culturally appropriate, culturally compelling and effective interventions for prevention and control of outdoor malaria transmission (Brick et al. [53])

Malaria prevention is viewed within the study community as a shared responsibility of the government and researchers. However, malaria prevention, control and elimination need a collective approach that includes the sustained used of vector control to prevent human-mosquito contact, treatment, and surveillance. In the study villages, increasing outdoor feeding by malaria vectors has been documented [34, 35, 44, 67, 68], but emphasis is still placed on the distribution and use of bed nets only which primarily prevent individual infection while sleeping indoors [69]. In order to move closer to elimination, however, there is a need to change the approaches to designing, implementing and evaluating interventions for malaria in Tanzania by taking into consideration the social and ecological aspects of continued and residual transmission. Policy-makers need to consider social, cultural and behavioural factors associated with risk of infection when designing malaria prevention strategies. One approach is to adapt the model developed by Bronfenbrenner [70], which was used in a study by Panter-Brick et al. [53], which contributed to identification of both social and cultural factors for risks in malaria, intervention use, community participation as well as reduction of malaria impact to the communty (Fig. 2). This explains that individual behaviour is influenced by environmental factors, including people’s social, institutional and cultural contexts and the beliefs and attitudes that influence and shape the social, political and economic conditions of a given society. The model helps to explain the importance of different parts of the system in a given society, and how its members relate and influence each other.

## Conclusion

People do not use interventions in the evening when outdoors during social and cultural practices and celebrations. Spending time outdoors to participate in social events, in accordance with cultural norms, expose people to mosquito bites and increase the risk of malaria infection. During such gatherings, the use of interventions to prevent bites is rare. Such gatherings not only provide opportunities for collaboration and contribute to cohesion, but also personal satisfaction and personal identity.

The increasing rate of outdoor feeding of the primary vectors of malaria in the study area, coincident with the use of outdoor areas for everyday activities and for ceremonial and social purposes, point to the need for effective interventions. There is over dependency on current interventions, primarily long lasting insecticide-impregnated bed nets, and single interventions should not be used as “one size fits all,” since transmission risks and intensity differ among areas [39]. Although respondents were aware of the risks of infection, further community education on the risks of outdoor malaria and the use of repellents may reduce negative perceptions on the interventions, such as repellents which is perceived to cause skin cancer [71] and increase the uptake of their use. The approach for malaria prevention needs to be culturally compelling [53], despite that area specific interventions, using a social-ecological model that incorporates social, cultural and environment factors, may appear to be expensive but will become inevitable as a strategy for malaria elimination [39]. Interventions are needed that are affordable and can be used during ceremonies and for general protection outdoors, complementing existing methods to prevent indoor transmission.

## Abbreviations

FGD: focus group discussion
IRS: insecticide residual spray
KII: key informant interview
LLINs: long-lasting insecticide nets
RDT: rapid diagnostic test
SSA: sub-Sahara Africa

## References

[1] WHO. World Malaria Report. Geneva: World Health Organization; 2016.

[2] Bhatt S, Weiss D, Cameron E, Bisanzio D, Mappin B, Dalrymple U (2015). The effect of malaria control on *Plasmodium falciparum* in Africa between 2000 and 2015. Nature.

[3] O’Meara WP, Mangeni JN, Steketee R, Greenwood B (2010). Changes in the burden of malaria in sub-Saharan Africa. Lancet Infect Dis..

[4] Ossè RA, Aïkpon R, Gbédjissi GL, Gnanguenon V, Sèzonlin M, Govoétchan R (2013). A shift from Indoor Residual Spraying (IRS) with bendiocarb to Long-Lasting Insecticidal (mosquito) Nets (LLINs) associated with changes in malaria transmission indicators in pyrethroid resistance areas in Benin. Parasit Vectors..

[5] Steketee RW, Campbell CC (2010). Impact of national malaria control scale-up programmes in Africa: magnitude and attribution of effects. Malar J..

[6] Hawley WA, Phillips-Howard PA, ter Kuile FO, Terlouw DJ, Vulule JM, Ombok M (2003). Community-wide effects of permethrin-treated bed nets on child mortality and malaria morbidity in western Kenya. Am J Trop Med Hyg.

[7] Killeen GF, Kihonda J, Lyimo E, Oketch FR, Kotas ME, Mathenge E (2006). Quantifying behavioural interactions between humans and mosquitoes: evaluating the protective efficacy of insecticidal nets against malaria transmission in rural Tanzania. BMC Infect Dis.

[8] Killeen GF, Smith TA, Ferguson HM, Mshinda H, Abdulla S, Lengeler C (2007). Preventing childhood malaria in Africa by protecting adults from mosquitoes with insecticide-treated nets. PLoS Med..

[9] Schellenberg J, Abdulla S, Nathan R, Mukasa O, Marchant TJ, Kikumbih N (2001). Effect of large-scale social marketing of insecticide-treated nets on child survival in rural Tanzania. Lancet.

[10] WHO. World Malaria Report. Geneva: World Health Organization; 2017.

[11] President Malaria Initiative (2018). Fighting malaria and saving lives.

[12] Ishengoma DS, Mmbando BP, Alifrangis M, Lemnge MM, Bygbjerg IC (2013). Declining burden of malaria over two decades in a rural community of Muheza district, north-eastern Tanzania. Malar J..

[13] Mmbando BP, Vestergaard LS, Kitua AY, Lemnge MM, Theander TG, Lusingu JP (2010). A progressive declining in the burden of malaria in north-eastern Tanzania. Malar J..

[14] Mtove G, Amos B, Nadjm B, Hendriksen IC, Dondorp AM, Mwambuli A (2011). Decreasing incidence of severe malaria and community-acquired bacteraemia among hospitalized children in Muheza, north-eastern Tanzania, 2006–2010. Malar J..

[15] Rutta AS, Francis F, Mmbando BP, Ishengoma DS, Sembuche SH, Malecela EK (2012). Using community-owned resource persons to provide early diagnosis and treatment and estimate malaria burden at community level in north-eastern Tanzania. Malar J..

[16] Prapanthadara L, Hemingway J, Ketterman AJ (1995). DDT-resistance in *Anopheles gambiae* (Diptera: Culicidae) from Zanzibar, Tanzania, based on increased DDT-dehydrochlorinase activity of glutathione S-transferases. Bull Entomol Res..

[17] Ranson H, N’Guessan R, Lines J, Moiroux N, Nkuni Z, Corbel V (2011). Pyrethroid resistance in African anopheline mosquitoes: what are the implications for malaria control?. Trends Parasitol..

[18] Trape J-F, Tall A, Diagne N, Ndiath O, Ly AB, Faye J (2011). Malaria morbidity and pyrethroid resistance after the introduction of insecticide-treated bednets and artemisinin-based combination therapies: a longitudinal study. Lancet Infect Dis..

[19] United Republic of Tanzania. Tanzania demographic and health survey and malaria indicator survey 2015–2016. Dar es Salaam, 2016.

[20] Kitau J, Oxborough RM, Tungu PK, Matowo J, Malima RC, Magesa SM (2012). Species shifts in the *Anopheles gambiae* complex: do LLINs successfully control *Anopheles arabiensis*?. PLoS ONE.

[21] Kouznetsov R (1977). Malaria control by application of indoor spraying of residual insecticides in tropical Africa and its impact on community health. Trop Doctor..

[22] Muirhead-Thomson R (1960). The significance of irritability, behaviouristic avoidance and allied phenomena in malaria eradication. Bull World Health Organ.

[23] Okumu FO, Kiware SS, Moore SJ, Killeen GF (2013). Mathematical evaluation of community level impact of combining bed nets and indoor residual spraying upon malaria transmission in areas where the main vectors are *Anopheles arabiensis* mosquitoes. Parasit Vectors..

[24] Martens W, Niessen LW, Rotmans J, Jetten TH, McMichael AJ (1995). Potential impact of global climate change on malaria risk. Environ Health Perspect.

[25] Lindblade KA, Walker ED, Onapa AW, Katungu J, Wilson ML (2000). Land use change alters malaria transmission parameters by modifying temperature in a highland area of Uganda. Trop Med Int Health.

[26] Patz JA, Graczyk TK, Geller N, Vittor AY (2000). Effects of environmental change on emerging parasitic diseases. Int J Parasitol.

[27] Kiware SS, Chitnis N, Moore SJ, Devine GJ, Majambere S, Merrill S (2012). Simplified models of vector control impact upon malaria transmission by zoophagic mosquitoes. PLoS ONE.

[28] Nkya TE, Akhouayri I, Poupardin R, Batengana B, Mosha F, Magesa S (2014). Insecticide resistance mechanisms associated with different environments in the malaria vector *Anopheles gambiae*: a case study in Tanzania. Malar J..

[29] Ranson H, Abdallah H, Badolo A, Guelbeogo WM, Kerah-Hinzoumbé C, Yangalbé-Kalnoné E (2009). Insecticide resistance in *Anopheles gambiae*: data from the first year of a multi-country study highlight the extent of the problem. Malar J..

[30] Ranson H, Lissenden N (2016). Insecticide resistance in African Anopheles mosquitoes: a worsening situation that needs urgent action to maintain malaria control. Trends Parasitol..

[31] Ferguson HM, Dornhaus A, Beeche A, Borgemeister C, Gottlieb M, Mulla MS (2010). Ecology: a prerequisite for malaria elimination and eradication. PLoS Med..

[32] Killeen GF (2013). A second chance to tackle African malaria vector mosquitoes that avoid houses and don’t take drugs. Am J Trop Med Hyg.

[33] Govella NJ, Chaki PP, Killeen GF (2013). Entomological surveillance of behavioural resilience and resistance in residual malaria vector populations. Malar J..

[34] Kaindoa EW, Matowo NS, Ngowo HS, Mkandawile G, Mmbando A, Finda M (2017). Interventions that effectively target *Anopheles funestus* mosquitoes could significantly improve control of persistent malaria transmission in south–eastern Tanzania. PLoS ONE.

[35] Lwetoijera DW, Harris C, Kiware SS, Dongus S, Devine GJ, McCall PJ (2014). Increasing role of *Anopheles funestus* and *Anopheles arabiensis* in malaria transmission in the Kilombero Valley, Tanzania. Malar J..

[36] Durnez L, Coosemans M. Residual transmission of malaria: an old issue for new approaches. In: Anopheles mosquitoes—New insights into malaria vectors (S. Manguin, Ed). Intech; 2013.

[37] Russell TL, Beebe NW, Cooper RD, Lobo NF, Burkot TR (2013). Successful malaria elimination strategies require interventions that target changing vector behaviours. Malar J..

[38] Zhu L, Müller GC, Marshall JM, Arheart KL, Qualls WA, Hlaing WM (2017). Is outdoor vector control needed for malaria elimination? An individual-based modelling study. Malar J..

[39] WHO. A framework for malaria elimination. Geneva: World Health Organization; 2017.

[40] Bayoh MN, Mathias DK, Odiere MR, Mutuku FM, Kamau L, Gimnig JE (2010). *Anopheles gambiae*: historical population decline associated with regional distribution of insecticide-treated bed nets in western Nyanza Province, Kenya. Malar J..

[41] Gillies MT, Furlong M (1964). An investigation into the behaviour of *Anopheles parensis* Gillies at Malindi on the Kenya coast. Bull Entomol Res.

[42] Reddy MR, Overgaard HJ, Abaga S, Reddy VP, Caccone A, Kiszewski AE (2011). Outdoor host seeking behaviour of *Anopheles gambiae* mosquitoes following initiation of malaria vector control on Bioko Island, Equatorial Guinea. Malar J..

[43] Renggli S, Mandike R, Kramer K, Patrick F, Brown NJ, McElroy PD (2013). Design, implementation and evaluation of a national campaign to deliver 18 million free long-lasting insecticidal nets to uncovered sleeping spaces in Tanzania. Malar J..

[44] Russell TL, Govella NJ, Azizi S, Drakeley CJ, Kachur SP, Killeen GF (2011). Increased proportions of outdoor feeding among residual malaria vector populations following increased use of insecticide-treated nets in rural Tanzania. Malar J..

[45] Russell TL, Lwetoijera DW, Maliti D, Chipwaza B, Kihonda J, Charlwood JD (2010). Impact of promoting longer-lasting insecticide treatment of bed nets upon malaria transmission in a rural Tanzanian setting with pre-existing high coverage of untreated nets. Malar J..

[46] Dunn CE, Le Mare A, Makungu C (2011). Malaria risk behaviours, socio-cultural practices and rural livelihoods in southern Tanzania: implications for bednet usage. Soc Sci Med.

[47] Adongo PB, Kirkwood B, Kendall C (2005). How local community knowledge about malaria affects insecticide-treated net use in northern Ghana. Trop Med Int Health.

[48] Atkinson JM, Fitzgerald L, Toaliu H, Taleo G, Tynan A, Whittaker M (2010). Community participation for malaria elimination in Tafea Province, Vanuatu: Part I Maintaining motivation for prevention practices in the context of disappearing disease. Malar J..

[49] Mazigo HD, Obasy E, Mauka W, Manyiri P, Zinga M, Kweka EJ, et al. Knowledge, attitudes, and practices about malaria and its control in rural northwest Tanzania. Malar Res Treatment. 2010;2010. 10.4061/2010/794261 PMC3275933 22332023

[50] Nieto T, Méndez F, Carrasquilla G (1999). Knowledge, beliefs and practices relevant for malaria control in an endemic urban area of the Colombian Pacific. Soc Sci Med.

[51] Harchut K, Standley C, Dobson A, Klaassen B, Rambaud-Althaus C, Althaus F (2013). Over-diagnosis of malaria by microscopy in the Kilombero Valley, Southern Tanzania: an evaluation of the utility and cost-effectiveness of rapid diagnostic tests. Malar J..

[52] Dunn CE, Le Mare A, Makungu C (2010). Malaria risk behaviours, socio-cultural practices and rural livelihoods in southern Tanzania: implications for bednet usage. Soc Sci Med.

[53] Panter-Brick C, Clarke SE, Lomas H, Pinder M, Lindsay SW (2006). Culturally compelling strategies for behaviour change: a social ecology model and case study in malaria prevention. Soc Sci Med.

[54] Bekker C, Rance W, Monteuuis O (2004). Teak in Tanzania. II. The Kilombero Valley Teak Company. Bois et forêts des Tropiques.

[55] Ogoma SB, Lweitoijera DW, Ngonyani H, Furer B, Russell TL, Mukabana WR (2010). Screening mosquito house entry points as a potential method for integrated control of endophagic filariasis, arbovirus and malaria vectors. PLoS Negl Trop Dis..

[56] Strauss A, Corbin J. Basics of qualitative research: Techniques and procedures for developing grounded theory. Sage Publications, Inc; 1998.

[57] Strauss A, Corbin JM (1997). Grounded theory in practice.

[58] Krueger RA (2014). Focus groups: a practical guide for applied research.

[59] Freitas H, Oliveira M, Jenkins M, Popjoy O. The Focus Group, a qualitative research method. JISRC Working Paper. 1998;010298.

[60] Weber RP (1990). Basic content analysis.

[61] Bringer JD, Johnston LH, Brackenridge CH (2006). Using computer-assisted qualitative data analysis software to develop a grounded theory project. Field Methods..

[62] Jens F. Traditional Music and Cultures of Kenya: Kikuyu- Circumcision. 2000–2003.

[63] A Culture of Circumcision in the Kurya Tribe of Tanzania. https://blog.compassion.com/circumcision-in-africa-a-culture-of-circumcision-in-the-kurya-tribe-of-tanzania/.

[64] 3 Meanings of Sunnah. http://aboutislam.net/shariah/hadith/hadith-faqs/3-meanings-sunnah/.

[65] Azar OH (2004). What sustains social norms and how they evolve? The case of tipping. J Econ Behav Organ.

[66] Monroe A, Harvey SA, Lam Y, Muhangi D, Loll D, Kabali AT (2014). “People will say that I am proud”: a qualitative study of barriers to bed net use away from home in four Ugandan districts. Malar J..

[67] Matowo NS, Moore J, Mapua S, Madumla EP, Moshi IR, Kaindoa EW (2013). Using a new odour-baited device to explore options for luring and killing outdoor-biting malaria vectors: a report on design and field evaluation of the Mosquito Landing Box. Parasit Vectors..

[68] Okumu FO, Sumaye RD, Matowo NS, Mwangungulu SP, Kaindoa EW, Moshi IR (2013). Outdoor mosquito control using odour-baited devices: development and evaluation of a potential new strategy to complement indoor malaria prevention methods. Malar World J..

[69] Programme National Malaria Control (2014). National malaria strategic plan 2014–2020.

[70] Bronfenbrenner U (1994). International encyclopedia of education.

[71] Moshi IR, Ngowo H, Dillip A, Msellemu D, Madumla EP, Okumu FO (2017). Community perceptions on outdoor malaria transmission in Kilombero Valley, Southern Tanzania. Malar J..

